# Non‐additive effects between genotypes: Implications for competitive fitness assays

**DOI:** 10.1002/ece3.10713

**Published:** 2023-11-07

**Authors:** Changyi Xiao, Sara Duarri‐Redondo, Dagny A. V. Thorhölludottir, Yiwen Chen, Christian Schlötterer

**Affiliations:** ^1^ Institut für Populationsgenetik Vetmeduni Vienna Austria; ^2^ Vienna Graduate School of Population Genetics Vienna Austria

**Keywords:** competitive fitness, *Drosophila*, experimental evolution, frequency‐dependent selection

## Abstract

Competitive fitness assays are widely used in evolutionary biology and typically rely on a reference strain to compare different focal genotypes. This approach implicitly relies on the absence of interaction between the competing genotypes. In other words, the performance of the reference strain must not depend on the competitor. This report scrutinized this assumption by competing diverged *Drosophila simulans* populations against a common reference strain. We detected strong evidence for interaction between the competing genotypes: (1) Frequency‐dependent selection was common with opposite effects in genetically diverged populations. (2) Temporal heterogeneity of fitness estimates, which can be partially attributed to a competitor‐specific delay in the eclosion of the reference strain. We propose that this inconsistent behavior of the reference strain can be considered a specific case of a genotype × environment interaction. Focal populations could modify the environment of the reference strain, either indirectly by altering the microbiome composition and food availability or directly by genotype‐specific cannibalism. Our results provide new insights into the interaction of diverged genotypes and have important implications for the interpretation of competitive fitness assays.

## Graphical abstract

It shows the frequency dependence of the number of offspring of the reference strain. We interpret this as evidence for interactions with the focal populations.

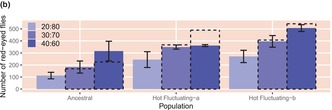

## 1 INTRODUCTION

Fitness is a fundamental concept in evolutionary biology where genotypes with higher fitness produce more offspring and consequently increase in frequency in the population. However, despite the simplicity of this concept, accurately quantifying fitness remains a significant empirical challenge. The hurdles for obtaining reliable empirical fitness estimates can be grouped into two categories: firstly, measuring fitness across the entire life cycle of an organism (Prout, 1965, 1971) and secondly, the uncertainty about the best approach to measure fitness (Haymer & Hartl, 1983).

Fitness can be assessed through either competitive or non‐competitive assays (Haymer & Hartl, 1983). Non‐competitive fitness estimates aim to quantify parameters that serve as proxies for fitness, with a focus on life history traits, such as the number of eggs/seeds or developmental time. Yet, indirect traits like body size and biomass have also been used. The conceptual problem of non‐competitive fitness lies in the assumption that comparison of absolute trait values can predict how different genotypes will perform in a competitive setting. Furthermore, for practical reasons fitness proxies are measured in a specific time interval, which can introduce biases and affect the accuracy of fitness estimates. For instance, the duration of female egg laying is limited to a fraction of the total fecundity period or the number of offspring is measured for a restricted time interval only. If the fecundity peak or developmental time differs between populations, this has strong effects on the fitness estimate although it is unclear whether these differences really translate in biologically relevant fitness differences. In contrast, relative fitness measurements make fewer implicit assumptions but, they also encounter several challenges, including frequency‐dependent selection (Curtsinger, 1990; DeBenedictis, 1977) and complex genotype× environment interactions (Burny et al., 2022; Fry, 2008; Fry et al., 1998; Kondrashov & Houle, 1994; Takano et al., 1987; Teotonio et al., 2002). As a result, competitive and non‐competitive fitness measurements do not always agree (Ayala, 1970; Haymer & Hartl, 1983).

In *Drosophila*, researchers measured competitive fitness by combining experimental evolution with balancer chromosomes to measure fitness of single chromosomes (Fowler et al., 1997; Gardner et al., 2005; Sved, 1975). Balancer chromosomes are a special tool in *Drosophila* genetics. With several inversions covering almost the entire chromosome recombination is suppressed, which allows measurements of allele frequency changes across multiple generations. Phenotypic markers allow the distinction of different chromosomes. Because frequency changes are determined across multiple generations, this approach provides reliable fitness measurements. In the absence of balancer chromosomes fitness estimates in outcrossing organisms are limited to single‐generation fitness measurements as recombination mixes genotypes.

The challenge for a wide‐spread use of competitive fitness assays is the availability of phenotypic markers which distinguish offspring from different parents. Because such markers are typically not available for the comparison of natural populations, competitive fitness assays rely on a reference strain with a phenotypic marker, which allows screening of many offspring. The use of reference strains for competitive fitness assays offers a significant advantage for comparing the fitness of strains/populations originating from different environments. Rather than comparing each strain/population against each other, it is possible to make an indirect comparison relative to the reference strain. For example, the relative fitness of five populations can be inferred by five competitive fitness assays with the same reference strain rather than with 10 pairwise fitness assays involving each possible combination of population pairs. Nevertheless, the success of competitive fitness assays with a reference strain critically depends on the absence of an interaction between genotypes. In other words, the performance of the reference strain must not be affected by the focal population.

In this study, we tested this assumption by asking whether the fitness of the reference strain depends on the competing population. We took advantage of experimental *Drosophila simulans* populations, which adapted for more than 100 generations to different temperature regimes in the laboratory. We used competitive fitness assays in which focal populations were competed against the same reference strain. We collected offspring over a broad time course to explore the reliability of fitness estimates across time. Our results are in agreement with previous findings indicating a significant frequency dependence on competitive fitness (Curtsinger, 1990; DeBenedictis, 1977). Additionally, we observed that fitness estimates vary with time. We attribute these uncertainties to the complex interactions of the reference strain with the focal populations.

## 2 METHODS

Fitness components of natural populations are frequently measured in the laboratory, where samples with a different adaptive history are compared at well‐defined culture conditions. While it is apparent that the laboratory cannot fully replicate natural conditions, this approach allows a tight control of focal environmental parameters, such as temperature. Here, we used polymorphic *D. simulans* populations, which evolved in different temperature regimes, but we measured their fitness in a hot fluctuating temperature regime. The focus was fitness of these populations in a temperature environment fluctuating between 18°C and 28°C. We were not interested in the influence of other differences in maintenance regime (e.g., timing of transfer to fresh food). Hence, we extended the observational period well beyond time interval of the experimental evolution maintenance regime.

Since we were interested to obtain fitness estimates that include most aspects of the life cycle, we allowed 100 mated females to lay eggs for 48 h. Hence, our fitness assays include fecundity and viability. We did not include sexual competition in our fitness assays to exclude confounding effects arising from pre‐mating reproductive isolation which was detected in some of the focal populations (Hsu et al., 2023). The interpretation of fitness can differ when sexual competition is not included; therefore, it is in general advisable to measure fitness across the entire life cycle of an organism (Prout, 1965, 1971). In our study, the inclusion of sexual competition would have only introduced an additional complication, which would have distracted from the key message, the complications of competitive fitness assays.

### 2.1 Experimental *D. simulans* populations

All experimental populations were founded from the same set of isofemale lines originating from a natural *D. simulans* population collected in Florida (Barghi et al., 2019).

1. Untreated: Reconstructed ancestral population established from the same isofemale lines that were used to start the selection experiments (19 lines were missing, because they have been lost during maintenance). The small population size of the isofemale lines strongly reduces the efficacy of selection and therefore very limited adaptation is expected during the maintenance. This expectation was confirmed in a comparison of ancestral and reconstructed ancestral populations, which show no allele frequency differences (Nouhaud et al., 2016). We refer to the untreated population as ancestral throughout the manuscript.
2. Hot fluctuating treatment (28/18°C): Populations were set up using 202 isofemale lines (Barghi et al., 2019). Five mated females from each isofemale line were used to establish the replicates. These replicates were kept in 12‐h light:12‐h dark cycles with a temperature of 28°C (light) and 18°C (dark) for about 240 generations.
3. Cold fluctuating treatment (20/10°C): Populations were established from the same isofemale lines as the replicate populations from the hot fluctuating treatment but maintained under a different temperature regime: 12‐h light:12‐h dark cycles with a temperature of 20°C (light) and 10°C (dark) for 115 generations.
4. Constant hot treatment (23°C): Populations were set up using 191 isofemale lines. Five mated females from each isofemale line were used to establish the replicates. Replicates were kept in 12‐h light:12‐h dark cycles with a constant temperature of 23°C for 173 generations.

All evolved populations were maintained at a census size of 1250 flies with a 50:50 sex ratio.

Hot fluctuating experimental evolution regime: In the hot fluctuating regime (28/18°C) flies have a generation time of approximately 2 weeks. Considering Day 1 as the day when flies start to eclose, flies that eclose between Day 1 and Day 4 will contribute to the next generation.

### 2.2 Phenotypic assays

Phenotypic assays were performed in two independent common garden experiments (CGE). Each experiment was performed in the hot fluctuating regime (28/18°C): 12‐h light:12‐h dark cycles with a temperature of 28°C (light) and 18°C (dark) with controlled egg density (400 eggs/bottle). All phenotypes were measured after two generations of the CGE to minimize transgenerational effects.

For phenotyping, we used up to five independently evolved populations from each evolutionary treatment. For the two CGEs, the ancestral population was reconstructed from the same isofemale lines that were also used to establish the experimentally evolved populations. Within each CGE, all populations were assayed on the same day to avoid potential uncontrolled temporal variation in each CGE. In both CGEs, we used technical replicates to account for sampling introduced by picking a subset of flies from the main focal population, as well as other technical factors.

#### 2.2.1 CGE1

Two populations evolved in a hot fluctuating treatment (237 generations) and an ancestral population were measured for relative fitness after two generations of common garden. For each population (Hot fluctuating‐a, Hot fluctuating‐b, and Ancestral), three replicates were measured.

After two generations of common garden, 3‐day‐old mated females (focal populations) were mixed with *D. simulans* w501 gravid females (reference population; https://www.ncbi.nlm.nih.gov/bioproject/PRJNA170244) and maintained in CGE conditions. Prior to mixing, w501 flies were maintained at room temperature, and covered a broader age distribution than the polymorphic population samples but had the same age distribution across measurements. Mated females were mixed with 3 different ratios (focal: reference): 20:80, 30:70, and 40:60, with a total of 100 females. After 24 h of CO_2_ recovery, mixed females were allowed to oviposit in a bottle for 48 h. Newly eclosed flies were collected daily for 13 days after the first fly eclosed. Although almost all flies were eclosed by Day 13, we included a final collection days later (Day 18), to collect the remaining flies.

#### 2.2.2 CGE2

Five hot fluctuating evolved (generation 241), five cold fluctuating evolved (generation 115), five hot constant evolved (generation 173), and one reconstructed ancestral population were phenotyped for relative fitness after two generations of CGE in the same fluctuating hot regime as described for CGE1. Each independently evolved population was phenotyped for all selection regimes, but jointly analyzed for the comparison of selection regimes.

For each evolved population, three replicates were created by merging 30 focal gravid females (4‐day to 6‐day old, the same age distribution in each bottle) with 70 w501 gravid females (4‐day to 9‐day old, the same age distribution in each bottle). The ancestral population had 15 replicates to match the evolved treatments. Prior to mixing, the w501 flies were maintained for multiple generations at room temperature. After 24 h of CO_2_ recovery, the mixed females oviposited in a bottle for 48 h in the hot fluctuating regime. The eclosed flies were collected daily over 10 days after the first fly eclosed. Based on results from CGE1, most flies eclosed during the first 10 days, and patterns between ancestral and evolved persist and do not change after Day 10.

### 2.3 Data analysis

All data analyses (including Fisher's exact test) were performed in R (v4.1.0) (R Core Team, 2018) and plots were done using ggplot2 package (v3.3.6) (Wickham, 2016). Since we were mostly interested to show the performance differences when different focal populations were used, rather than specific comparisons, we plot the 95% confidence intervals and consider non‐overlapping intervals as distinct trajectories. Hence, the “differences” mentioned in the main text reflect to non‐overlapping confidence intervals, rather than specific tests. When we compare time points explicitly, the tests are mentioned explicitly.

The data from CGE2 were fitted into a generalized linear mixed model using the glmer() function from lme4 package in R (Bates et al., 2015). The proportion of focal flies (red‐eyed) was treated as the response that follows a binomial distribution with the total number of flies given as weights, treatments were considered as fixed effects and populations within the treatment as random effects. An extra observation level random effect was fitted into the model to correct for overdispersion. The proportion of focal flies (red‐eyed) until Day 5 (*λ* = 0.171) and Day 10 (*λ* = 0.102) were fitted into the model separately. Significance tests were performed with the package emmeans (v 1.7.5) (Lenth, 2022), and multiple testing correction (FDR) was performed on all *p*‐values across the two models. The alpha level was set at 0.05 to determine significance.

## 3 RESULTS

In the absence of phenotypic markers, which distinguish evolved and ancestral populations or independently evolved replicate populations, measuring competitive fitness using a reference strain with a phenotypic marker are a natural choice. We used an inbred reference strain with white eyes (w501) in pairwise competition with multiple polymorphic populations, which evolved in different temperature regimes (Table 1). Since our focal populations show pre‐mating reproductive isolation (Hsu et al., 2023), we did not include sexual competition in our fitness assays. Our fitness estimates combined the fitness components fecundity and viability.

**TABLE 1:** Overview of the two common garden experiments (CGE).

CGE	Focal populations (number of technical replicates)^a^
CGE1	1 Ancestral (3), 2 hot fluctuating (3).
CGE2	5 Ancestral (3), 5 hot fluctuating (3), 5 cold fluctuating (3), 5 hot constant (3).

^a^ Populations refer to the replication of the experimental evolution (or establishment of the ancestor) and the common garden treatment. Technical replicates refer to independent measurements of the same population (but different flies) for the relative fitness assays.

### 3.1 Frequency dependence of competitive fitness estimates

We used different ratios of mated females from the focal populations (20%, 30%, 40%) and the white‐eyed reference (80%, 70%, 60%) for egg laying over 2 days (i.e., the same number of flies were used in each experiment). Three genetically distinct populations, the ancestral population, and two independently evolved populations were tested in a hot fluctuating environment (28/18°C).

18 days after the first fly eclosed, the total number of eclosed flies was similar for all three ratios, although some differences among the three genetically diverged focal populations could be detected (Figure 2a). The influence of the different ratios was more apparent when the offspring of the focal and reference population were analyzed separately. In the absence of frequency‐dependent selection, twice as many red‐eyed flies are expected with 40% rather than 20% of females from the focal group. For all three focal populations, the 40% experiment differed from the expected factor of two (Figure 2b). While the two evolved populations produced fewer offspring than expected, the opposite trend was seen for the ancestral population: more offspring eclosed at 40% than expected from the number of focal eclosed flies at 20%.

**FIGURE 1:**
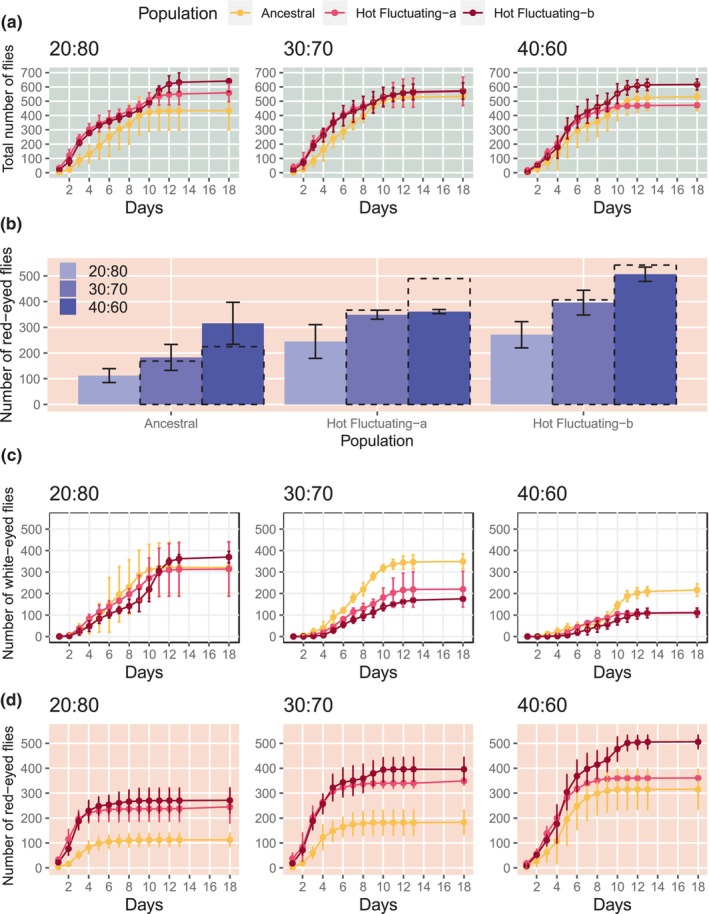
Competitive fitness is frequency‐dependent (data from CGE1). (a) Time‐resolved cumulative number of total eclosed flies (white‐eyed and red‐eyed). (b) The number of red‐eye flies eclosed between Day 1 and Day 18. The number of red‐eyed flies is shown in colored bars, while the dashed line indicates the expectation based on the number of red‐eyed flies from the experiments with 80% white‐eyed reference population. Error bars represent 95% CI. (c) Time‐resolved eclosure of the white‐eyed reference flies for the three different ratios. Error bars represent the 95% CI. (d) Time‐resolved eclosure of the red‐eyed reference flies for the three different ratios. Error bars represent 95% CI. The color of the lines distinguishes the focal populations in the competition and the background color indicates the genotype of the displayed flies: total number of flies (red‐eyed and white‐eyed: light green), red‐eyed flies only (light red), white‐eyed flies only (white).

A striking pattern was observed for the white‐eyed reference strain w501. The white‐eyed reference always eclosed later than the focal populations, but the delay was less pronounced for the competition with ancestral flies than with evolved flies (Figure 1c). Furthermore, with a decreasing fraction of white‐eyed females, the time of eclosion was even more delayed. In competition with hot‐evolved flies, the eclosion of white‐eyed flies was delayed by about 5 days at a 40:60 ratio compared with the 20:80 ratio (Figure 1c): the number of eclosed white‐eyed flies from Days 1 to 5 and from Days 6 to 10 differed for these two ratios (Fisher's exact test *p*‐value for Fluctuating‐a = 1.94e‐13 and Fluctuating‐b = 1.34e‐20, Table S1). This pattern clearly demonstrates that the presence of the red‐eyed focal flies retards the development of the reference strain and that the delay depends on the competitor and the ratio of reference to focal population during egg laying. The three different ratios did not only affect the eclosion time but also the total number of eclosed white‐eyed flies. While the number of white‐eyed offspring did not differ in the competition with the three different reference populations at the ratio 20:80, at the ratio 40:60 more white‐eyed flies eclosed in competition with the ancestral population.

### 3.2 The critical importance of time

We monitored the total number of eclosed flies for up to 18 days (Figure 1a) and observed different eclosion trajectories for focal and reference populations (Figure 1c,d). The number of eclosed flies plateaued later for white‐eyed than for red‐eyed flies. At least to some extent, this difference can be attributed to the delayed eclosion of the white‐eyed flies (Figure 1c,d). This implies that fitness measured relative to the reference will depend critically on the time at which the number of eclosed flies is monitored (see Figure S2 for time‐resolved relative fitness).

### 3.3 Population‐specific effects

CGE1 already indicated that the interaction between reference and competitor could affect fitness estimates. In the second experiment (CGE2), we explored how this interaction is modified when the focal populations are genetically distinct (as a consequence of adaptation to different environments). We included populations adapted to four different evolutionary treatments: an untreated ancestral population, cold treatment fluctuating between 10°C and 20°C for 115 generations, hot constant treatment at 23°C for 173 generations, and hot fluctuating treatment fluctuating between 18°C and 28°C for 241 generations. Within each experimental treatment, we included five independently evolved populations, each with three replicates. 15 replicates of the ancestral population were used to match the evolved populations. The competitive fitness assays were carried out in a hot environment fluctuating between 18°C and 28°C using a ratio of 70% reference females and 30% females from the focal evolutionary treatment.

**FIGURE 2:**
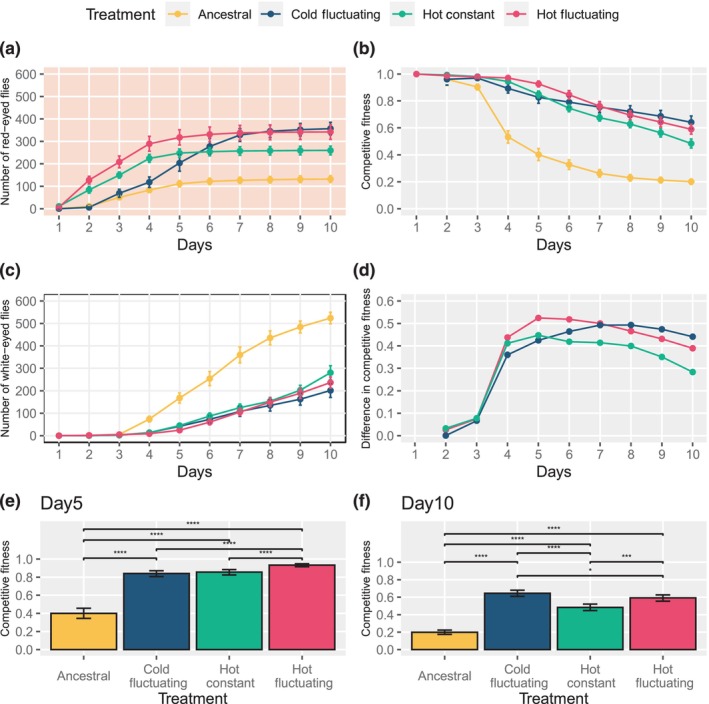
Time‐dependent fitness differences among flies evolved under different treatments (data from CGE2). (a) Time‐resolved cumulative number of red‐eyed flies from different evolutionary treatments. (b) Time‐resolved relative fitness (Competitive fitness = Number of red‐eyed flies/Total number of flies). (c) Time‐resolved eclosion of white‐eyed flies in competition with flies from different evolutionary treatments. (d) Difference in competitive fitness estimates. The three experimental treatments are compared to the ancestral flies (mean competitive fitness of evolved flies—mean competitive fitness of ancestral flies). The color code matches the evolved population that is compared to the ancestral population. (e) Competitive fitness estimate based on the proportion of red‐eyed flies eclosed between Day 1 and Day 5. (f) Competitive fitness estimate based on the proportion of red‐eyed flies eclosed between Day 1 and Day 10. Error bars represent 95% CI. Colors indicate the treatment; background colors indicate different fitness estimates based on the number of red‐eyed flies (light red), the number of white‐eyed flies (white), and the proportion of red‐eyed flies (light gray). Only significant comparisons are shown, and the significance levels are indicated as * (<.05), ** (<.01), *** (<.001), **** (<.0001). Note that in panel 2B, the data point for Day 1 is missing for Ancestral and Cold populations, as no flies had eclosed. In panel 2D, the difference is measured from Day 2, as there is no data available for the Ancestral population for Day 1.

Populations that evolved independently under the same experimental treatment had a very similar number of offspring (similar to CGE1 at 30:70 ratio) and were jointly analyzed for CGE2. Flies from the two hot temperature treatments (23°C and 18/28°C) eclosed faster than flies from the other treatments (Figure 2a). Until Day 5, populations from the hot fluctuating treatment produced most offspring (Figure 2a). The ancestral population had the fewest offspring, independent of the time point at which the offspring were counted (Figure 2a). This pattern is fully consistent with the expected fitness differences, and this also holds for competitive fitness estimates (Figure 2b), although the differences between the evolved populations were more pronounced for the number of red‐eyed flies. Flies from the hot fluctuating treatment were better adapted than flies from the hot constant and ancestral treatment. Thus, the population which evolved in the same temperature regime which was used to measure fitness, produced most offspring. The cold‐evolved flies, however, had very interesting time‐dependent dynamics. During the first days of eclosion, they behaved similarly to the ancestral flies and had fewer offspring than hot‐evolved flies. Around Day 4, however, an increasing number of cold‐evolved flies eclosed, such that after Day 6 more cold‐evolved flies had eclosed than constant hot‐evolved flies (Figure 2a). At Day 10 the number of cold‐evolved flies was even similar to the ones that evolved in the hot fluctuating treatment. The time‐dependent pattern observed for red‐eyed flies (Figure 2a) also holds for competitive fitness estimates as evidenced by the differences between Days 5 and 10 (Figure 2e,f). Interestingly, the cold fluctuating population on Day 10 performed significantly better than the hot fluctuating populations (Figure 2f). Hence, competitive fitness measurements at different time points could result in entirely different conclusions about the fitness of cold‐evolved and hot‐evolved populations.

Similar to CGE1, the eclosion of the white‐eyed flies was more delayed in experiments with evolved flies than those with the ancestral population, which also produced the largest number of white‐eyed offspring (Figure 2c). At all time points the differences among white‐eyed flies from the three different experimentally evolved populations were quite small. Only at the end of the experiment the fewest white‐eyed offspring were observed in competition with the cold‐evolved flies.

## 4 DISCUSSION

In this study, we used the same reference strain either in different frequency and/or in combination with different competitors. Our analyses showed that the eclosure behavior of the reference population was strongly affected by the competitor and frequency, suggesting interaction effects among larvae from the two competing populations. In total, we gathered four different pieces of evidence for interaction between competing populations. (1) The eclosion time of white‐eyed flies was delayed to a different extent in experiments using different frequencies of the two competing populations. (2) The proportion of white‐eyed flies among experiments differed more than expected by the mixture ratios (Figure S1). (3) The eclosion time of white‐eyed flies differed between competition experiments with ancestral and evolved flies. (4) The frequency dependence of the white‐eyed flies was highly dependent on the competitor.

Genotype × environment interactions are common (e.g., Bochdanovits & de Jong, 2003; Horvath & Kalinka, 2016; Huang et al., 2020; Nguyen et al., 2017) and have even been described in the form of environmentally triggered differences in genome‐wide selection responses (Burny et al., 2022; Rudman et al., 2022). We hypothesize that the non‐additive effects in our study may reflect an altered environment caused by the competing population. Altering the ratio of focal and reference flies as well as the competition with populations adapted to different treatments could modify the competition environment, despite other components of the experiment, in particular, culture conditions (e.g., food, temperature, and light) remain the same. We propose four possible drivers that could modify the environment in our competition experiments. (1) microbiome. It is well‐understood that the microbiome composition changes during temperature adaptation and even replicate populations can differ (Mazzucco & Schlötterer, 2021). Changing the ratio of competing flies or using flies with a different evolutionary history will modify the microbiome in the competition experiment. Since the microbiome composition changes the fitness (Rudman et al., 2019) and physiology (Martino et al., 2017; Matos & Leulier, 2014; Shin et al., 2011) of the flies, it is conceivable that the non‐additive interactions in our experiment are driven by the microbiome composition. (2) cannibalism. An alternative, but not mutually exclusive, explanation is population‐specific larval cannibalism. Larvae have been shown to distinguish between genetically distinct eggs and preferentially cannibalize eggs with different ancestry (Narasimha et al., 2019). Furthermore, the CHC composition has evolved during adaptation to a hot fluctuating environment (Hsu et al., 2023). Hence, it is conceivable that larvae cannibalize preferentially genetically distinct larvae. The retarded eclosion of the white‐eyed offspring fits the cannibalism hypothesis. Because evolution during high larval density leads to the evolution of cannibalism (Vijendravarma et al., 2013) and the populations evolved at high larval density, they are expected to show more cannibalism than ancestral one. The cannibalism hypothesis matches the stronger delay in the eclosion of white‐eyed flies when competing with evolved populations than with the ancestral one. Nevertheless, it does not explain why the ancestral population shows more cannibalism than the reference strain. Furthermore, the frequency‐dependent eclosure behavior of the white‐eyed offspring would require a non‐linear genotype‐specific cannibalism. (3) small differences in developmental rate between flies from different treatments may result in differential access to fresh food without waste products. As lower quality food further delays development (Botella et al., 1985), this could amplify the initial differences and eventually result in the different suppression of eclosion of white‐eyed flies. We further anticipate that different adaptation to poor food quality could also explain the time‐dependent variation in competitive fitness. (4) Genotype‐by‐density interactions were previously observed for developmental time in *D. melanogaster* (Horvath & Kalinka, 2016), which implies that response to crowding varies among strains. Hence, it is possible that the different fecundities of the focal genotypes (Barghi et al., 2019; Mallard et al., 2018) changes larval density, which in turn affects the performance of the white‐eyed strain.

The frequency‐dependent fitness effects seen in CGE1 and the dependence on the time point when fitness is measured indicate substantial interactions between competing populations. We illustrate the influence of these interactions on the inference of fitness differences between two focal populations. Rather than comparing two focal populations directly, we make an indirect comparison: the competitive fitness estimates (with the same reference strain) are compared against each other. Each line depicts the difference in fitness between one evolved population and the ancestral population (Figure 2d). It is apparent that the fitness difference varies between time points and the crossing of the lines indicates a change in rank order. Hence, our results have important consequences for the interpretation of competitive fitness estimates.

As a note of caution, we need to emphasize that we used only a single reference strain. While it is possible that other reference strains would not show this pattern, our primary focus was to shed light on the implications of genotype‐by‐genotype interactions for relative fitness measurements. Our goal was not to explore differences among alternative reference strains with respect to variation in the strengths of effects and the fraction of reference strains that show genotype‐by‐genotype interactions for relative fitness. Nevertheless, similar results have been previously reported (Adell et al., 1990). The fitness component viability of two wild‐type strains cannot be predicted from their competition against reference strains. This suggests that our findings of non‐additivity are not contingent on the choice of reference strain.

Our study adds to the ample literature on the complexity of fitness measurements. We propose that competing populations are creating a specific environment, which depends on the frequency of the reference and/or the differentiation of the two competing populations. It is not yet clear whether the difference between populations is driven by a diverged microbiome, population‐specific cannibalism, larval crowding, or a hitherto unknown process. Nevertheless, if our hypothesis is substantiated by further studies, we caution that results from competitive fitness assays need to be evaluated against the effects of the changing environment. Our study added the importance of time‐dependent heterogeneity of fitness components to the list of well‐understood problems of fitness estimates. We propose that reliable fitness measurements need to account for the evolution of temporal patterns as well as for the possibility of population‐specific modifications of the environment.

## AUTHOR CONTRIBUTIONS

**Changyi Xiao:** Data curation (equal); formal analysis (equal); investigation (equal); visualization (equal); writing – review and editing (supporting). **Sara Duarri‐Redondo:** Data curation (equal); formal analysis (equal); investigation (equal); visualization (equal); writing – review and editing (supporting). **Dagny A. V. Thorhölludottir:** Investigation (equal); writing – review and editing (supporting). **Yiwen Chen:** Investigation (equal); writing – review and editing (supporting). **Christian Schlötterer:** Conceptualization (equal); funding acquisition (lead); writing – original draft (lead); writing – review and editing (lead).

## Supporting information


Appendix S1

## Data Availability

All data and scripts are available at Zenodo: https://doi.org/10.5281/zenodo.8382826.

## References

[ece310713-bib-0001] Adell, J. C. , Moya, A. , Molina, V. , & Gonzalez‐Candelas, F. (1990). On the analysis of viability data: An example with Drosophila. Heredity, 65(Pt 1), 39–46. 2120154 10.1038/hdy.1990.67

[ece310713-bib-0002] Ayala, F. J. (1970). Population fitness of geographic strains of Drosophila‐Serrata as measured by interspecific competition. Evolution, 24, 483. 28562996 10.1111/j.1558-5646.1970.tb01783.x

[ece310713-bib-0003] Barghi, N. , Tobler, R. , Nolte, V. , Jaksic, A. M. , Mallard, F. , Otte, K. A. , Dolezal, M. , Taus, T. , Kofler, R. , & Schlötterer, C. (2019). Genetic redundancy fuels polygenic adaptation in *Drosophila* . PLoS Biology, 17, e3000128. 30716062 10.1371/journal.pbio.3000128 PMC6375663

[ece310713-bib-0004] Bates, D. , Mächler, M. , Bolker, B. , & Walker, S. (2015). Fitting linear mixed‐effects models using lme4. Journal of Statistical Software, 67, 1–48.

[ece310713-bib-0005] Bochdanovits, Z. , & de Jong, G. (2003). Experimental evolution in *Drosophila melanogaster*: Interaction of temperature and food quality selection regimes. Evolution, 57, 1829–1836. 14503624 10.1111/j.0014-3820.2003.tb00590.x

[ece310713-bib-0006] Botella, L. M. , Moya, A. , Gonzalez, M. C. , & Mensua, J. L. (1985). Larval stop,delayed development and survival in overcrowded cultures of *Drosophila melanogaster* – Effect of urea and uric‐acid. Journal of Insect Physiology, 31, 179–185.

[ece310713-bib-0007] Burny, C. , Nolte, V. , Dolezal, M. , & Schlötterer, C. (2022). Genome‐wide selection signatures reveal widespread synergistic effects of two different stressors in *Drosophila melanogaster* . Proceedings of the Royal Society B: Biological Sciences, 289, 20221857. 10.1098/rspb.2022.1857 PMC9579754 36259211

[ece310713-bib-0008] Curtsinger, J. W. (1990). Frequency‐dependent selection in Drosophila – Estimation of net fitness in Pseudohaploid populations. Evolution, 44, 857–869. 28569019 10.1111/j.1558-5646.1990.tb03810.x

[ece310713-bib-0009] Debenedictis, P. A. (1977). Studies in the dynamics of genetically variable populations. I. Frequency‐ and density‐dependent selection in experimental populations of *Drosophila melanogaster* . Genetics, 87, 343–356. 17248767 10.1093/genetics/87.2.343 PMC1213745

[ece310713-bib-0010] Fowler, K. , Semple, C. , Barton, N. H. , & Partridge, L. (1997). Genetic variation for total fitness in *Drosophila melanogaster* . Proceedings of the Royal Society B: Biological Sciences, 264, 191–199. 10.1098/rspb.1997.0027 PMC1688253 9061969

[ece310713-bib-0011] Fry, J. D. (2008). Genotype‐environment interaction for total fitness in Drosophila. Journal of Genetics, 87, 355–362. 19147925 10.1007/s12041-008-0058-7

[ece310713-bib-0012] Fry, J. D. , Nuzhdin, S. V. , Pasyukova, E. G. , & Mckay, T. F. C. (1998). QTL mapping of genotype‐environment interaction for fitness in *Drosophila melanogaster* . Genetics Research, 71, 133–141. 10.1017/s0016672398003176 9717436

[ece310713-bib-0013] Gardner, M. P. , Fowler, K. , Barton, N. H. , & Partridge, L. (2005). Genetic variation for total fitness in *Drosophila melanogaster*: Complex yet replicable patterns. Genetics, 169, 1553–1571. 15545656 10.1534/genetics.104.032367 PMC1449528

[ece310713-bib-0014] Haymer, D. S. , & Hartl, D. L. (1983). The experimental assessment of fitness in *Drosophila*. II. A comparison of competitive and noncompetitive measures. Genetics, 104, 343–352. 17246139 10.1093/genetics/104.2.343 PMC1202080

[ece310713-bib-0015] Horvath, B. , & Kalinka, A. T. (2016). Effects of larval crowding on quantitative variation for development time and viability in *Drosophila melanogaster* . Ecology and Evolution, 6, 8460–8473. 28031798 10.1002/ece3.2552 PMC5167028

[ece310713-bib-0016] Hsu, S.‐K. , Lai, W.‐Y. , Novak, J. , Lehner, F. , Jakšić, A. M. , Versace, E. , & Schlötterer, C. (2023). Two different adaptive speciation mechanisms operate during adaptation to a novel hot environment. *bioRxiv*.

[ece310713-bib-0017] Huang, W. , Carbone, M. A. , Lyman, R. F. , Anholt, R. R. H. , & Mackay, T. F. C. (2020). Genotype by environment interaction for gene expression in *Drosophila melanogaster* . Nature Communications, 11, 5451. 10.1038/s41467-020-19131-y PMC7595129 33116142

[ece310713-bib-0018] Kondrashov, A. S. , & Houle, D. (1994). Genotype‐environment interactions and the estimation of the genomic mutation rate in *Drosophila melanogaster* . Proceedings of the Royal Society B: Biological Sciences, 258, 221–227. 10.1098/rspb.1994.0166 7886063

[ece310713-bib-0019] Lenth, R. V. (2022). *emmeans: Estimated marginal means*, *aka least squares means* . R package version 1.7.5.

[ece310713-bib-0020] Mallard, F. , Nolte, V. , Tobler, R. , Kapun, M. , & Schlötterer, C. (2018). A simple genetic basis of adaptation to a novel thermal environment results in complex metabolic rewiring in *Drosophila* . Genome Biology, 19, 119. 30122150 10.1186/s13059-018-1503-4 PMC6100727

[ece310713-bib-0021] Martino, M. E. , Ma, D. , & Leulier, F. (2017). Microbial influence on *Drosophila* biology. Current Opinion in Microbiology, 38, 165–170. 28668769 10.1016/j.mib.2017.06.004

[ece310713-bib-0022] Matos, R. C. , & Leulier, F. (2014). Lactobacilli‐host mutualism: “learning on the fly”. Microbial Cell Factories, 13(Suppl 1), S6. 25186369 10.1186/1475-2859-13-S1-S6 PMC4155823

[ece310713-bib-0023] Mazzucco, R. , & Schlötterer, C. (2021). Long‐term gut microbiome dynamics in *Drosophila melanogaster* reveal environment‐specific associations between bacterial taxa at the family level. Proceedings of the Royal Society B: Biological Sciences, 288, 20212193. 10.1098/rspb.2021.2193 PMC8670958 34905708

[ece310713-bib-0024] Narasimha, S. , Nagornov, K. O. , Menin, L. , Mucciolo, A. , Rohwedder, A. , Humbel, B. M. , Stevens, M. , Thum, A. S. , Tsybin, Y. O. , & Vijendravarma, R. K. (2019). *Drosophila melanogaster* cloak their eggs with pheromones, which prevents cannibalism. PLoS Biology, 17, e2006012. 30629594 10.1371/journal.pbio.2006012 PMC6328083

[ece310713-bib-0025] Nguyen, N. H. , Hamzah, A. , & Thoa, N. P. (2017). Effects of genotype by environment interaction on genetic gain and genetic parameter estimates in red tilapia (*Oreochromis* spp.). Frontiers in Genetics, 8, 82. 28659970 10.3389/fgene.2017.00082 PMC5468391

[ece310713-bib-0026] Nouhaud, P. , Tobler, R. , Nolte, V. , & Schlötterer, C. (2016). Ancestral population reconstitution from isofemale lines as a tool for experimental evolution. Ecology and Evolution, 6, 7169–7175. 27895897 10.1002/ece3.2402 PMC5114691

[ece310713-bib-0027] Prout, T. (1965). The estimation of fitnesses from genotypic frequencies. Evolution, 19, 546–555.

[ece310713-bib-0028] Prout, T. (1971). The relation between fitness components and population prediction in Drosophila. I: The estimation of fitness components. Genetics, 68, 127–149. 17248524 10.1093/genetics/68.1.127 PMC1212576

[ece310713-bib-0029] R Core Team . (2018). R: A language and environment for statistical computing. R Foundation for Statistical Computing.

[ece310713-bib-0030] Rudman, S. M. , Greenblum, S. , Hughes, R. C. , Rajpurohit, S. , Kiratli, O. , Lowder, D. B. , Lemmon, S. G. , Petrov, D. A. , Chaston, J. M. , & Schmidt, P. (2019). Microbiome composition shapes rapid genomic adaptation of *Drosophila melanogaster* . Proceedings of the National Academy of Sciences of the United States of America, 116, 20025–20032. 31527278 10.1073/pnas.1907787116 PMC6778213

[ece310713-bib-0031] Rudman, S. M. , Greenblum, S. I. , Rajpurohit, S. , Betancourt, N. J. , Hanna, J. , Tilk, S. , Yokoyama, T. , Petrov, D. A. , & Schmidt, P. (2022). Direct observation of adaptive tracking on ecological time scales in *Drosophila* . Science, 375, eabj7484. 35298245 10.1126/science.abj7484 PMC10684103

[ece310713-bib-0032] Shin, S. C. , Kim, S. H. , You, H. , Kim, B. , Kim, A. C. , Lee, K. A. , Yoon, J. H. , Ryu, J. H. , & Lee, W. J. (2011). *Drosophila* microbiome modulates host developmental and metabolic homeostasis via insulin signaling. Science, 334, 670–674. 22053049 10.1126/science.1212782

[ece310713-bib-0033] Sved, J. A. (1975). Fitness of third chromosome homozygotes in *Drosophila melanogaster* . Genetical Research, 25, 197–200. 810388 10.1017/s0016672300015603

[ece310713-bib-0034] Takano, T. , Kusakabe, S. , & Mukai, T. (1987). The genetic‐structure of natural‐populations of *Drosophila melanogaster* .20. Comparison of genotype‐environment interaction in viability between a northern and a southern‐population. Genetics, 117, 245–254. 3117620 10.1093/genetics/117.2.245 PMC1203201

[ece310713-bib-0035] Teotonio, H. , Matos, M. , & Rose, M. R. (2002). Reverse evolution of fitness in *Drosophila melanogaster* . Journal of Evolutionary Biology, 15, 608–617.

[ece310713-bib-0036] Vijendravarma, R. K. , Narasimha, S. , & Kawecki, T. J. (2013). Predatory cannibalism in *Drosophila melanogaster* larvae. Nature Communications, 4, 1789. 10.1038/ncomms2744 23653201

[ece310713-bib-0037] Wickham, H. (2016). ggplot2 elegant graphics for data analysis. Springer Verlag.

