# Supplementary material for: Non‐additive effects between genotypes: Implications for competitive fitness assays
Source: Ecol Evol. 2023 Nov 7;13(11):e10713. doi: 10.1002/ece3.10713 (PMC10630047; doi:10.1002/ece3.10713)
Supplement: Supplementary file 1 — Appendix S1 [file ECE3-13-e10713-s001.docx]

Supplementary material

Supplementary Figure 1. The number of white-eye flies eclosed between day 1 and day 18 in CGE1. The observed number of white-eyed flies are shown in colored bars, while the dashed bar indicates the expectation based on the average number of white-eyed flies based on the 80% reference population. Error bars represent the 95% CI.

Supplementary Figure 2. Time-resolved relative fitness (Competitive fitness = Number of red-eyed flies/Total number of flies) in CGE1. Error bars represent the 95% CI.

Supplementary Table1 Fisher exact test of number of white-eyed flies eclosed from day 1 to 5 versus from day 6 to day 10 between two ratios for populations measured in CGE1.

**Ancestral**

|  | Ratio 20:80 | Ratio 40:60 |
| --- | --- | --- |
| Day 1 to Day 5 | 263 | 117 |
| Day 6 to Day 10 | 670 | 316 |

#Fisher exact test p-value = 0.70

**Fluctuating-a**

|  | Ratio 20:80 | Ratio 40:60 |
| --- | --- | --- |
| Day 1 to Day 5 | 343 | 60 |
| Day 6 to Day 10 | 474 | 253 |

#Fisher exact test p-value = 1.94e-13

**Fluctuating-b**

|  | Ratio 20:80 | Ratio 40:60 |
| --- | --- | --- |
| Day 1 to Day 5 | 247 | 17 |
| Day 6 to Day 10 | 411 | 212 |

#Fisher exact test p-value = 1.34e-20
